# Factors influencing choice of care-seeking for acute fever comparing private chemical shops with health centres and hospitals in Ghana: a study using case–control methodology

**DOI:** 10.1186/s12936-016-1351-1

**Published:** 2016-05-25

**Authors:** Evelyn K. Ansah, Margaret Gyapong, Solomon Narh-Bana, Constance Bart-Plange, Christopher J. M. Whitty

**Affiliations:** Research and Development Division, Ghana Health Service, P.O. Box MB-190, Accra, Ghana; National Malaria Control Programme, Ghana Health Service, Accra, Ghana; London School of Hygiene & Tropical Medicine, London, UK

**Keywords:** Drug retail shop, Fever, Chemical shop, Malaria, Health centre, Hospital, ACT, Diagnosis, Targeting

## Abstract

**Background:**

Several public health interventions to improve management of patients with fever are largely focused on the public sector yet a high proportion of patients seek care outside the formal healthcare sector. Few studies have provided information on the determinants of utilization of the private sector as against formal public sector. Understanding the differences between those who attend public and private health institutions, and their pathway to care, has significant practical implications. The chemical shop is an important source of care for acute fever in Ghana.

**Methods:**

Case–control methodology was used to identify factors associated with seeking care for fever in the Dangme West District, Ghana. People presenting to health centres, or hospital outpatients, with a history or current fever were compared to counterparts from the same community with fever visiting a chemical shop.

**Results:**

Of 600 patients, 150 each, were recruited from the district hospital and two health centres, respectively, and 300 controls from 51 chemical shops. Overall, 103 (17.2 %) patients tested slide positive for malaria. Specifically, 13.7 % (41/300) of chemical shop patients, 30.7 % (46/150) health centre and 10.7 % (16/150) hospital patients were slide positive. While it was the first option for care for 92.7 % (278/300) chemical shop patients, 42.7 % (64/150) of health centre patients first sought care from a chemical shop. More health centre patients (61.3 %; 92/150) presented with fever after more than 3 days than chemical shop patients (27.7 %; 83/300) [AOR = 0.19; p < 0.001 CI 0.11–0.30]. Although the hospital was the first option for 83.3 % (125/150) of hospital patients, most (63.3 %; 95/150) patients arrived there over 3 days after their symptoms begun. Proximity was significantly associated with utilization of each source of care. Education, but not other socioeconomic or demographic factors were significantly associated with chemical shop use.

**Conclusions:**

The private drug retail sector is the first option for the majority of patients, including poorer patients, with fever in this setting. Most patients with fever arrive at chemical shops with less delay and fewer signs of severity than at public health facilities. Improving chemical shop skills is a good opportunity to diagnose, treat or refer people with fever early.

## Background

Acute febrile illness is the commonest syndrome presenting in most African healthcare settings in both the formal public health sector and the informal private sector [1]. A high proportion, and often the majority, of those who seek care do not do so in the formal healthcare sector [2]. It is well recognized for example that the majority of children and pregnant women with malaria do not access formal health care [3]. In many African countries, self-treatment with drugs from general and chemical shops is reported widely and ranges from 4 to 87 % of cases [4–6]. Private drug retail shops are often closer, in terms of location, to where the users live and the opportunity costs of using them are sometimes lower than for the public sector, even when this is free [7]. Studies in Malawi and Ghana found that for a higher proportion of people or caregivers with fever, their first option was to obtain drugs over the counter from a drug retail or chemical shop [8, 9]. Filmer, in his review of fever and its treatment in sub-Saharan Africa, also found that in rural areas, treatment for children in the richest quintile is about 30 % more likely to be sought from a pharmacy or a shop than for children in the poorest quintile [10].

A Ghanaian study found that generally people do not adopt a single treatment-seeking pattern for uncomplicated malaria. While some visit health facilities immediately they feel unwell, others visit drug stores to purchase any drug they deem appropriate for the disease suspected or mention their health condition to the vendor who then decides on which drug is most appropriate. Some others also initiate treatment at home with some left over drugs or herbal preparations. A combination of two or more options could be adopted concurrently [11].

Factors associated with seeking care at a health facility include user fees, transportation to the facility, opportunity costs of lost time at income generating activities, cost of medicines and hospital supplies, as well as negative experiences at overcrowded and understaffed health facilities and difficulties encountered while navigating a hospital visit [9, 12]. Reasons why some people would chose to go to a health facility include services rendered, availability of laboratory services in the health facilities, the use of health insurance as a mode of financing services, proximity to the health facility, lack of knowledge of the cause of their disease condition and not getting cured after self-medication or seeking treatment at the drug store [11]. For example, urban care givers may be more likely to choose home treatment, shop or hospital treatment compared to no or traditional care, relative to rural [11].

The important role drug retailers play at community level, however, has been well acknowledged and several interventions have been developed to improve the delivery of appropriate malaria treatments through these retailers, including in Ghana [13–15]. Some studies have also shown that making even partially efficacious treatments available more rapidly closer to home has been associated with measurable reductions in severe morbidity and overall child mortality, including for malaria [16, 17]. This is important because many interventions in the public health sector to target fever case management affect only those who attend public sector outlets and will have limited or no effect on those who do not attend them.

As malaria-endemic countries in Africa use the relatively more expensive artemisinin-containing anti-malarial drugs, and other causes of potentially severe febrile illnesses become progressively more important with the fall in malaria incidence, defining clearly the role of private sector drug retailers has become even more important [18]. Whilst some studies have documented the important role that drug stores can play in care-seeking behaviour [19–21], few studies have provided information on the determinants of utilization of the private sector as against formal public sector care in a way which allows accurate comparison, or controlling for major potential confounding factors. Understanding the differences between those who attend public and private health institutions, and their pathway to care, has significant practical implications and it is important to understand where patients with fever go to seek care, and why in order to improve targeting of interventions.

This study, therefore, using case control methodology, aimed to identify factors associated with care seeking for fever in the main private sector drug outlet in rural settings in Ghana (chemical shops) compared to two types of public sector health facilities. The chemical shops, which are licensed and regulated by the Ghana Pharmacy Council, sell over the counter medicines. The two types of health facilities are the district hospital, which provides primary, inpatient referral care and health centers, which provide basic outpatient primary care.

In the context of availability of rapid diagnostic tests for testing and artemisinin combination therapy for the treatment of malaria in chemical shops in a rural and semi-rural area similar to many parts of West Africa, this study aimed to examine socioeconomic status and distance as factors in healthcare-seeking behaviour, and what proportion of cases, including potentially severe cases, had previously sought care in another sector, as well as associations with malaria. It compared those who go to the most common private provider (chemical shops), with those who go to health centres and hospital outpatients—the two standard forms of formal public healthcare provider.

## Methods

### Study site and population

The study was carried out in the Dangme West District of Ghana, a rural district with an estimated 2012 population of 130,570 based on the 2010 census. Most of the population lives in scattered small communities of less than 2000 people. Vehicular transport is unavailable in many parts of the district and people have to walk long distances (approx. 2–6 h) to reach the nearest main road making access to formal care difficult. The district is typical of poor disadvantaged rural districts across the country. Poverty is widespread. Health services are delivered from one district hospital, four health centres, 13 Community Health and Planning Services (CHPS) compounds and five private facilities. The public health facilities are at three levels of the district health system. The lowest level (the community level) is where the Community Health Officer (CHO) operating from the Community Health Planning and Service (CHPS) compound provides basic curative and preventive services. The middle level is the Health centre where a Medical Assistant and five or more other staff provide curative and preventive services. The final level is the District Hospital with a doctor in charge providing a wider range of services with admission facilities.

The district hospital and three of the health centres are located in the four largest towns in the district. A total of 56 private chemical shops and six pharmacies also sell pharmaceutical products. Earlier studies carried out in this setting showed that for presumed “malaria” in the household (in practice febrile illness), the first action taken in order of the most common were home treatment, chemical shop, health centre, hospital, drug peddler, and traditional healer in that order [22–24]. Recent Demographic surveillance data from the district indicated that about 67 % of all deaths took place at home [25].

### Study design

The study used a case–control methodology and analysis to identify factors associated with attending different healthcare settings with fever, although the outcome is the source of care for a fever comparing hospital or health centre (public sector) with chemical shops (private sector) rather than a disease. The health centre arm comprised two health centres; Ningo and Prampram health centres while the district hospital formed the hospital arm. Patients who presented to either of the two health centres, or at the district hospital with a complaint of fever or a history of fever over the preceding 48 h (public sector patient) were selected after informed consent. A counterpart from the same community visiting a chemical shop at around the same time to seek care for fever was identified from the records collected at a chemical shop (private sector patient) and became a control. The control needed to be identified within the 5 days following the visit of the case to the health facility. The first patient fulfilling these criteria that visits any of the shops is selected. If no match is identified within 5 days, the public sector patient was dropped from the study analysis.

Patients of any age, residing in the district who reported at any of the participating health facilities or chemical shops with complaints of fever during the period of the study were potentially eligible for inclusion. Pregnant women, severely ill patients requiring immediate referral and non-residents of the district were excluded. Details of the patients and directions to their homes were taken for the purpose of follow-up for an interview at home. The information and consent form indicated that patients recruited into the study would be contacted at home later.

A structured questionnaire was used to ascertain factors that could influence care-seeking behaviour of patients with fever at community level including some demographic variables, preventive practices, and socio-economic status (Table 1). As part of the on-going trial, research blood slides were taken for each client recruited in participating chemical shops for later double reading. Similarly, cases recruited in any of the health facilities had a blood slide taken for later double reading at the hospital by experienced microscopists since malarial fever is one of the commonest and important causes of fever. Malaria was defined as fever or history of fever with parasitaemia >2500 parasites/µl for both cases and controls. An asset index was constructed based on previously validated questions [26]. Study participants were ranked by socioeconomic status using quintiles.

**Table 1:** Potential factors influencing care-seeking for fever investigated

Category	Factors
Demographic	Age
	Sex
Preventive practices	Insecticide-treated net (ITN) ownership
	Insecticide-treated net (ITN) use
Socio-economic status	Patient’s highest educational level or highest educational level of caregiver (in case of a minor)
	Income
	Occupation
	Type of lighting or cooking fuel
	Main source of water
	Type of toilet facility
	Type of roof, wall and floor material
	Ownership of house
	Ownership of household gadgets such as TV, refrigerator, phone, mobile phone radio etc.
	Ownership of vehicle
	Ownership of income-generating equipment
	Ownership of farm and type
	Wealth quintile
	Rural or urban residence

### Statistical methods and power calculation

The study was powered to detect at least a 20 % absolute difference (20–40 %) between the chemical shop arm and either the health centre or the hospital for post-primary educational level as a proxy for socioeconomic status. The total sample size the study team aimed to recruit was 600 comprised of 150 health centre patients, 150 hospital patients and a total of 300 patients from the chemical shops allowing for some failure to match.

The data was double entered into EPI Data version 3.10 followed by validation of the data entered. Analysis was carried out in Stata version 11. Analysis of the data involved the calculation of odds ratios (ORs) and exact confidence intervals (CIs) using Stata SE (version 11) comparing chemical shop patients and health centre patients, and comparing chemical shop patients and hospital outpatients. Logistic regression models were used to assess the association of various factors with care-seeking for fever at a chemical shop compared to the two public sector settings with all the major factors in the same model. Factors that could be potentially associated with care-seeking for fever were pre-specified (Table 1).

### Ethical considerations

Ethical approval was obtained from the Ethics Review committee of the Ghana Health Service (GHS) in Ghana (GHS-ERC 01/5/10). The main ongoing clinical trial through which the chemical shop clients had been recruited was registered on the Clinical Trials site (Clinicaltrials.gov NCT01907672). Permission was sought from the Dangme West District Health Management Team and the Health Facility Management Teams for this study. Informed consent was also sought from all study participants before their inclusion in the study. Study records were identified by means of study IDs.

## Results

Between November 22, 2012 and January 30, 2013, a total of 600 patients were recruited into the study comprised of 150 patients from the two health centres, 150 from the hospital and 300 from chemical shops. Out of the 62 shops that sold anti-malarials in the district, 53 participated in this study, with 51 shops contributing controls. Patients seeking care at the health centre and hospital differed in demographic, socio-economic, and preventive practices against malaria among others from those who sought care at a chemical shop. Detailed results and odds ratios are presented in Tables 2 and  3.

**Table 2:** Estimated odds ratio for factors influencing health-seeking for acute fever in chemical shops as compared to health centres

Factors associated with care-seeking for acute fever	No (%) chemical shop N = 300	No (%) health centre N = 150	Crude OR	p value	Adjusted OR [95 % CI]	p value
			Chemical shop vs health centre [95 % CI]			
Age less than 5 years	64 (21.3 %)	45 (30.0 %)	0.63 [0.40–0.99]	0.04	0.62 [0.38–1.01]	0.06
Female gender	147 (49.0 %)	94 (62.7 %)	0.57 [0.38–0.86]	0.006	0.69 [0.45–1.10]	0.10
Patient reported severity	98 (32.7 %)	67 (44.7 %)	0.60 [0.40–0.90]	0.01	0.73 [0.47–1.10]	0.10
Delay to treatment (sought care after 3 days)	83 (27.7 %)	92 (61.3 %)	0.24 [0.16–0.37]	<0.001	0.19 [0.11–0.30]	<0.001
Lives near a health centre	60 (20.0 %)	64 (42.7 %)	0.33 [0.22–0.52]	<0.001	0.21 [0.12–0.39]	<0.001
In the poorest two quintiles	110 (36.7 %)	79 (52.7 %)	0.52 [0.35–0.77]	0.01	0.71 [0.44–1.10]	0.15
Has no post primary education	130 (43.3 %)	116 (77.3 %)	0.22 [0.14–0.35]	<0.001	0.24 [0.14–0.41]	<0.001
Slept under an ITN the previous night	95 (31.7 %)	67 (44.7 %)	0.57 [0.38–0.86]	0.007	0.65 [0.40–1.05]	0.08

**Table 3:** Estimated odds ratio for factors influencing health-seeking for acute fever in chemical shops as compared to hospitals

Factors associated with care-seeking for acute fever	No (%) chemical shop	No (%) hospital	Crude OR	p value [95 % CI]	Adjusted OR	p value
			Chemical shop vs hospital			
Age less than 5 years	64 (21.3 %)	24 (16 %)	1.42	0.18 [0.85–2.40]	1.65	0.08 [0.94–2.87]
Female gender	147 (49.0 %)	97 (64.7 %)	0.52	0.02 [0.35–0.79]	0.49	0.002 [0.31–0.77]
Patient reported severity	98 (32.7 %)	68 (45.3 %)	0.59	0.009 [0.39–0.87]	0.56	0.016 [0.35–0.90]
Delay to treatment (sought care after 3 days)	83 (27.7 %)	95 (63.3 %)	0.22	<0.001 [0.15–0.34]	0.29	<0.001 [0.18–0.46]
Lives near a hospital	120 (40.0 %)	115 (76.7 %)	0.16	0.877 [0.64–1.69]	0.12	<0.001 [0.06–0.24]
In the poorest two quintiles	110 (36.7 %)	79 (52.7 %)	0.52	<0.001 [0.35–0.77]	0.71	0.15 [0.44–1.14]
Has no post primary education	130 (43.3 %)	86 (57.3 %)	0.57	0.005 [0.38–0.85]	0.32	<0.001 [0.19–0.54]
Slept under an ITN the previous night	95 (31.7 %)	48 (32.0 %)	0.98	0.94 [0.65–1.50]	0.81	0.40 [0.50–1.31]

### Demographic characteristics of patients

#### Health centre compared to chemical shop

There was little difference in the age of those presenting at chemical shops versus health centres, by mean age (19.9 vs 22.2 years, respectively), however, in children under 5 years of age with fever presenting for care, a higher proportion presenting at the health centre [30 %; 45/150] as compared to the chemical shop [21.3 %; 64/300] were children. A lower proportion of the patients who sought care at the chemical shop were females [49.0 %; 147/300] as compared to those who did so from the health centre [62.7 %; 94/150]. This was not significant after adjusting for a number of factors including age, sex, and distance of residence from health facility [AOR = 1.45; p = 0.09 95 % CI 0.94–2.24].

#### Hospital compared to chemical shop

Patients who sought care at the hospital were of a mean age of 22.2 years just like those who sought care at the health centre and though generally older than those who sought care in a chemical shop (19.9 years), the difference in age was not significant. There was also no significant difference in the proportion of children less than 5 years of age with fever presenting for care at the chemical shop (21.3 %; 64/300) as compared to the hospital (16 %; 24/150) [AOR = 1.65; p = 0.08 95 % CI 0.94–2.87]. A significantly lower proportion of females sought care at a chemical shop (49.0 %; 147/300) compared to the hospital outpatients (64.7 %; 97/150) [AOR = 0.49; p = 0.002 CI 0.31–0.77].

### Socio-economic status of patients

#### Health centre compared to chemical shop

Educational level was strongly associated with accessing health care at the chemical shop. The proportion of patients with no post primary education seeking care for fever at the chemical shop was 43.3 % (130/300) as against 77.3 % (116/150) of those who sought care at the health centre [AOR = 0.24; p < 0.001 CI 0.14–0.41]. Those who sought care for fever from the health centre were also in general, poorer than those who did so from the chemical shop [52.7 %; 79/150] versus [36.7 %; 110/300] for the poorest two quintiles. This was, however, not significant after adjusting for a number of factors such as age, sex, and distance of residence from the health facility [AOR = 0.71; p = 0.15 CI 0.44–1.10]. Overall, 38.7 % (58/150) of health centre patients indicated that they lived in a rural area and this was not significantly different from chemical shop patients [32.3 %; 97/300] (p = 0.183).

#### Hospital compared to chemical shop

The patients who sought care at the hospital were also significantly less well educated (57.3 %; 86/150) than those seeking care for fever at the chemical shop (43.3 %; 130/300) [AOR = 0.32; p < 0.001 CI 0.19–0.54]. Although the hospital patients presenting with fever were less poorer in terms of the lowest two quintiles (34.0 %; 51/150) than those who presented at the chemical shop (36.7 %; 110/300), this was not significant after adjusting for known confounding factors [AOR = 1.04; p = 0.88 CI 0.64–1.69]. The proportion of hospital patients who lived in a rural area [26.0 %; 39/150] was not significantly different from that of chemical shop patients [32.3 %; 97/300] (p = 0.168).

### Distance from health facility and health-seeking behaviour

#### Health centre compared to chemical shop

Overall, 42.7 % (64/150) of those who sought care for fever from the health centre lived near one, as compared to only 20 % (60/300) of those who sought care for fever at a chemical shop living close to a health centre [AOR = 0.21; p < 0.001 CI 0.12–0.39] suggesting proximity was a major factor in seeking care from health centres. Living near a health facility in this study was defined as living within 5 kms of the health facility. Only 7.3 % (11/150) of patients who sought care for fever at a health centre lived near a hospital but chose to visit a health centre. Of the 150 health centre patients, 30 % (45/150) indicated that in order to access the health centre they had walked, while one said he had used a bicycle. The remainder accessed the health centre by using some form of vehicular transport. In contrast, a very high proportion of patients who accessed care for fever at the chemical shop [87 %; 261/300] had walked there. Two used a bicycle whilst the remaining patients used some form of vehicular transport.

#### Hospital compared to chemical shop

Of the 150 patients who sought care for fever at a hospital, 76.7 % (115/150) lived close to the hospital as compared to 40 % (120/300) of patients who sought care for fever at a chemical shop. In contrast, none of the patients who sought care for fever at a hospital lived close to a health centre. Of the 150 hospital patients, 32 % (48/150) indicated that they had accessed the hospital by walking from their homes whilst the rest used some form of vehicular transport. This is in contrast to 87 % (261/300) chemical shop patients who walked to the shop where they sought care. Patients who lived near the hospital were more likely to seek care for fever from there (76.7 %; 115/150) than from a chemical shop (40.0 %; 120/300) [AOR = 0.12; p < 0.001 CI 0.06–0.24].

### Order of choice of options for healthcare for fever

The health centre was the first option for care for 39.3 % (59/150) of patients utilizing health centre services as compared to 83.3 % (125/150) of hospital patients. For patients seeking care for fever at the chemical shop, it was the first option for 92.7 % (278/300) with fever. Of the 150 health centre patients, 42.7 % (64/150) had visited the chemical seller as their first option for care for the current fever episode. Other first options that had been previously sought by health centre fever patients prior to arriving at the health centre included use of left over medicines at home [10.7 %; 16/150], visit to a health facility [4.7 %; 7/150], use of herbal preparations [2.0 %; 3/150] and purchase of medicines from the drug peddler [0.7 %; 1/150] respectively.

Of the 150 hospital patients, 6.0 % (9/150) had already sought care for the current fever episode from a chemical shop while 10.7 % (16/150) had sought care at another health facility as first options. Additionally, 15.3 % (23/150) had visited two further places after their first option prior to arriving at the hospital whilst two had visited one other place for health care for the same fever episode.

Overall, 2.3 % (7/300) of the patients attending the chemical shop had visited another shop earlier suggesting this is a rare practice. Other options of care utilized among chemical shop fever patients included the use of left over medicines at home [2.7 %; 8/300], visit to a health facility [1.7 %; 5/300], and use of herbal preparations [0.7 %; 2/300] respectively. Overall, 58.5 % (351/600) of all patients who participated in the study used the chemical shop as a first option for care for fever.

### Time between onset of symptoms and access to healthcare

The proportion of patients who sought care more than 3 days after their symptoms begun was lower among the chemical shop patients [27.7 %; 83/300] than among the health centre patients [61.3 %; 92/150] [AOR = 0.19; p < 0.001 CI 0.11–0.30]. Patients seeking care for fever at a chemical shop were more likely to do so earlier after the onset of symptoms than those who sought care at the health centre. Overall only 12 % (18/150) of health centre patients sought care within 24 h of onset of symptoms as compared to 26.3 % (79/300) of chemical shop patients. Although the hospital was the first option for 83.3 % (125/150) of those who sought care there, most (63.3 %; 95/150) patients still arrived there more than 3 days after their symptoms begun as compared to the chemical shop (27.7 %; 83/300). [AOR = 0.29; p < 0.001 CI 0.18–0.46]. In contrast to chemical shops, fewer health centre patients sought care within 24 h of onset of symptoms [26.3 %; 79/300] versus [12.0 %; (18/150)]. Overall, 35.3 % (53/150) hospital patients arrived there within 24 h of onset of symptoms.

Among those who were seeking care at any of the places as a first option, 66.1 % (39/59) and 61.6 % (77/125) of health centre and hospital patients respectively arrived more than 3 days after the onset of symptoms as compared to only 26.6 % (74/278) of chemical shop patients who did so.

### Perceived severity and health-seeking behaviour of patients

Analysis was restricted to the 233 patients who had reported moderately severe disease, defined as those who had lethargy or convulsions as reported by respondents. Patterns were very different between the three healthcare settings. Among patients with fever and symptoms compatible with severe disease visiting the health centres 73 % (49/67) had sought care somewhere else prior to arrival, and 53 % (36/67) had previously done so from a chemical shop. Restricting this to the poorest two quintiles 61 % (23/38) had first sought care at a chemical shop.

Among the hospital and chemical shop patients with symptoms compatible with severe disease, only 17.7 % (12/68) and 16.3 % (16/98) of this sub-group of patients respectively had sought care elsewhere prior to arrival.

### Preventive practices for malaria and health-seeking behaviour

Ownership of an insecticide-treated net (ITN) was generally high among all patients seeking health care for fever in the three places. Overall 72.7 % (109/150) of patients utilizing the health centre owned ITNs as compared to 67.3 % (202/300) of patients who sought care for fever from a chemical shop. With regards to use of the ITNs, a higher proportion of patients attending a health centre (44.7 %; 67/150) slept under an ITN the previous night as compared to those who sought care at a chemical shop (31.7 %; 95/300) but the difference was not significant [AOR = 0.65; p = 0.08 CI 0.40–1.05].

Among patients who sought care at the hospital, 62.0 % (93/150) owned ITNs. Although those patients who engaged in at least one preventive practice for malaria such as sleeping under an ITN the previous night were less likely to seek care at a chemical shop as compared to a hospital, this was not significant [AOR = 0.81; p = 0.40 CI 0.50–1.31]. See Table 3.

### Prevalence of malaria parasitaemia

Overall, 103 (17.2 %) of patients involved in the study tested positive for malaria when slides were taken. Of the total number of chemical shop patients 13.7 % (41/300) were positive for malaria parasites as compared to 30.7 % (46/150) of health centre patients [OR = 0.36 p = <0.001 CI 0.22–0.58]. Among the hospitals patients only 10.7 % (16/150) were positive for malaria by blood slide.

## Discussion

In malaria and other potentially severe febrile illness, the decision by patients and caregivers on where and when to seek care is a complex one involving among other things, cost (direct and indirect), distance, and perceived severity of disease. This study demonstrates that there were substantially more delays, and a higher proportion of patients perceived as severe in health centres compared to chemical shops. A high proportion of those attending health centres had first sought care at a chemical shop, and there was more delay in people reaching a health centre for care after the onset of symptoms as compared to chemical shops. This included patients who had self-reported moderately severe disease, most of whom had previously been to chemical shops in the private sector, and also those in the lowest wealth quintiles. The results showed a clear trend for less formal education to be associated with using the public sector. Interactions with other measures of socioeconomic status were complex, and poorer patients who self reported severe disease were more likely to have visited chemical shop first. Although those who sought care for fever from the health centre and hospital were also, in general, poorer than or as poor as those who did so from the chemical shop, the difference was not significant in this study. Studies in three other African countries found that the likelihood of seeking care from a commercial source (pharmacy/shop) was over three times higher in the richest as compared to the poorest quintile [10].

Recent global efforts have concentrated on improving healthcare in the formal healthcare setting including in Ghana, both at the hospital and health centre, and in particular in improving malaria diagnosis using rapid diagnostic tests [27–29]. These efforts do not address the period before patients reach formal healthcare or those who exclusively use the private sector. Two interventions, which are not mutually exclusive, could lead to earlier correct treatment. One is to reduce delay before reaching the formal public sector, which is challenging to achieve. The second is to strengthen diagnostic capacity and correct treatment at the private sector drug outlets (here chemical shops) where patients present first. It is often assumed either that the poorest use the public sector, or that the poorest only use the closest care. This study shows the reality is more complex; whilst there is a tendency for those going first to chemical shops to be richer and have more education, poorer patients with severe disease attending a health centre would have first sought care at a chemical shop. Of patients attending chemical shops with fever, a higher proportion had malaria than those attending hospitals. Only a relatively small proportion of self-reported moderately severe cases had malaria parasites, highest in health centres and lowest in hospital settings, which generally serve urban populations. Therefore, putting all the effort into improving malaria case management at the hospital without involving the chemical shop will not be optimal; interventions to help the poorest need to take account of this reality. The study makes it clear that in any setting, presumptive treatment solely for malaria would not be appropriate as the majority, including the majority of severe patients, do not have the disease. This is in keeping with findings from elsewhere in Africa and Asia [30, 31].

Some patients with severe disease seem to attend hospital directly; this may have been associated with proximity or with an assessment of the severity of the illness by the caregivers themselves. Proximity appeared to be an important factor in the choice of source of care. Additionally, this study showed that patients who engaged in at least one preventive practice for malaria were less likely to seek care at a chemical shop.

Previous studies have shown that because of the pluralistic health care system in many settings, patients move in between care providers depending on what they think is wrong with them and where they think they can get the best care. In African settings, a condition may either be considered suitable for a health facility, a church, a traditional healer or a spiritualist depending on the perceived cause of the problem. Patients either move between providers or use some of them simultaneously until the condition is resolved. The health facility, depending on socio economic status, perceived quality of care and distance may be a last resort or when the condition is very severe [32–34]. However this study showed that the chemical seller was the first point of call for most cases of fever including those with symptoms of severity since over half of those with symptoms compatible with severity had visited the chemical shop first. This was not dissimilar from what was found in other studies both in Ghana and other parts of Africa [8, 9].

This study has limitations. The selection of controls for cases does not allow a complete comparison between hospitals and health centres, only between each of these and the chemical shops. The measure of severity is a self-assigned one based on two easy to determine syndromes, rather than clinician-assigned WHO severity, although verbal autopsy data suggest this is a reasonable proxy [35]. In common with most other studies, asset index and education level data are only a rough approximation for socioeconomic factors; this is likely to dilute socioeconomic effects. Case–control design, of which this is a variant, depends critically on choice of cases and controls and there is always a danger of over-matching. Although there are other factors that might affect the choice of outlet type such as reputation of provider, expected quality of care, costs, etc., these factors, which are mostly based on the patients’ perception, were not the focus of this study and this may, therefore, be a limitation. Since the effects seen here are sufficiently large, and consistent, they are unlikely to be misleading.

## Conclusions

The private retail sector is an important source of care for fever at the community level. It remains the first option for the majority of patients with fever in this setting. Most patients with fever arrive there much earlier than at any health facility. The interaction between poverty and which sector is attended is complex. Patients either move between providers or use some of them simultaneously till the condition is resolved. In this context chemical shops see a higher proportion of malaria cases than the hospital, although lower than health centres. It is important to improve the quality of care for fever in the private sector and strengthen its linkage with the formal health care sector if the needed intervention is to be instituted early enough to reduce morbidity and mortality from malaria.

## Abbreviations

CHPS: Community Health and Planning Services
ID: identification
ITN: insecticide-treated net
WHO: World Health Organization
